# Nephroprotective Role of *Beta vulgaris* L. Root Extract against Chlorpyrifos-Induced Renal Injury in Rats

**DOI:** 10.1155/2019/3595761

**Published:** 2019-11-22

**Authors:** Gadah Albasher, Rafa Almeer, Saud Alarifi, Saad Alkhtani, Manal Farhood, Fatimah O. Al-Otibi, Noorah Alkubaisi, Humaira Rizwana

**Affiliations:** ^1^King Saud University, Department of Zoology, College of Science, Riyadh, Saudi Arabia; ^2^King Saud University, Department of Botany and Microbiology, College of Science, Riyadh, Saudi Arabia

## Abstract

Organophosphorus pesticides (OPs) are widely used for agricultural and housekeeping purposes. Exposure to OPs is associated with the progression of several health issues. Antioxidant agents may be powerful candidates to minimise adverse reactions caused by OPs. The aim of the present study was to evaluate the nephroprotective effects of red beetroot extract (RBR) against chlorpyrifos- (CPF-) induced renal impairments. CPF induced kidney dysfunction, as demonstrated by changes in serum creatinine and urea levels. Moreover, CPF exposure induced oxidative stress in the kidneys as determined by increased malondialdehyde and nitric oxide levels, decreased glutathione content, decreased catalase, superoxide dismutase, glutathione peroxidase, and glutathione reductase activities, and decreased nuclear factor (erythroid-derived 2)-like-2 factor expression. In addition, CPF induced inflammation in renal tissue as evidenced by increased release of tumor necrosis factor-alpha and interleukin-1*β* and upregulation of inducible nitric oxide synthase. Furthermore, CPF promoted cell death as demonstrated by decreased Bcl-2 and increased Bax and caspase-3 levels. Treatment with RBR one hour prior to CPF treatment blocked the effects observed in response to CPF alone. Our results suggest that RBR could be used to alleviate CPF-induced nephrotoxicity through antioxidant, anti-inflammatory, and antiapoptotic activities.

## 1. Introduction

Contamination with pesticides has emerged as a serious problem worldwide. Chlorpyrifos (O,O-diethyl O-3,5,6-trichloro-2-pyridyl phosphorothioate, CPF) is a moderately persistent broad-spectrum organophosphorus pesticide extensively used in cultivation due to its effectiveness in insect and worm control. CPF principally acts as an acetylcholinesterase (AChE) inhibitor [1]. AChE is found mainly at neuromuscular junctions and terminates neurotransmission of ACh. However, CPF was shown to target the immune and antioxidant defence systems [2]. Due to the widespread use of CPF, humans may be exposed to CPF directly or indirectly. The main routes of exposure to CPF are through consumption of contaminated foods, inhalation, and adsorption through the skin during preparation and application. Exposure to CPF induces several pathological conditions including neurotoxicity, endocrine disturbance, reproductive toxicity, immunological perturbations, and hepatorenal injury in both animals and humans. Exposure to CPF reportedly elicits toxicity via several mechanisms including generation of reactive oxygen species (ROS), production of proinflammatory cytokines such as tumor necrosis factor-alpha (TNF-*α*) and interleukin-1 beta (IL-1*β*), and induction of apoptosis [3].

*B. vulgaris* (also known as beetroot) is a plant member of the *Amaranthaceae* family (formerly placed in the *Chenopodiaceae* family). It is extensively cultivated worldwide, particularly in subtropical and tropical countries in Africa and in Asia, and in Mediterranean countries [4]. The roots contain a number of minerals including K, Cu, Mg, Zn, Ca, P, and Na, vitamins, and phytochemicals such as polyphenols and carotenoids. Unlike other red plants that contain anthocyanin pigments, the red/purple colour of beetroot is due to the presence of betalain pigments such as betacyanins and betaxanthins [5]. Beetroot has been used in folk medicine due to its vasodilatory, antihypertensive, antidiabetic, hepatoprotective, antioxidant, anti-inflammatory, and anticarcinogenic properties [6]. Furthermore, beetroot has also been shown to increase athletic performance [7].

The aim of this study was to evaluate the potential benefits of red beetroot methanolic extract (RBR) against CPF-induced nephrotoxicity by evaluating oxidative status, inflammation, apoptosis, and renal histological alterations in male rats.

## 2. Materials and Methods

CPF was purchased from a pesticide and chemical company located in Riyadh, KSA. Prior to administration, CPF was diluted with distilled water (dH_2_O) to a final concentration of 10 mg CPF/3.5 ml·H_2_O (w/v). Fresh red beetroot was obtained from a local market in Riyadh, KSA in November 2018. The plant material was identified and authenticated by a botanist (Botany Department, College of Science, King Saud University, Riyadh, KSA). The roots were washed with running tap water and ground into small pieces using an electrical blender. The methanolic extract of RBR was prepared by macerating the obtained juice and particles three times in aqueous methanol (70%) for 48 h at a ratio of 1 : 10 (w/v) at 4°C. The extract was filtered, and the organic solvent was removed by vacuum evaporation followed by lyophilisation. The obtained RBR was stored at −80°C until further analysis.

### 2.1. Experimental Design

Twenty-eight adult male Wistar albino rats (11 weeks old; 140–170 g) were housed five per cage in the animal facility of the Zoology Department, College of Science, King Saud University (KSA) under controlled conditions of 22–24°C and 40–60% relative humidity with a normal light/dark cycle. All experiments were performed in accordance with the Guidelines of the National Program for Science and Technology of the Faculty of Science, King Saud University. The study protocols were approved by the Ethics Committee of King Saud University (Riyadh, KSA; H-01-R-059).

After one week of acclimation, the rats were divided randomly into four groups (*n* = 7) and gavaged with the indicated treatments once daily for 28 consecutive days. The treatment groups were as follows: control group: received physiological saline solution (0.9% NaCl); RBR group: administered RBR at a dose of 300 mg/kg; CPF group: received CPF solution at a dose of 10 mg/kg; and RBR + CPF group: received 300 mg/kg RBR 1 h prior to administration of 10 mg/kg CPF. CPF dosing was performed according to the method outlined by Peiris and Dhanushka [8]. The oral dose of RBR was selected based on a preliminary experiment using three doses of RBR: 100, 200, and 300 mg/kg. This preliminary study showed that oral administration of RBR at a dose of 300 mg/kg effectively minimised CPF-induced nephrotoxicity (data not shown). Twenty-four hours after the final treatment, the rats were sacrificed under anaesthesia. The kidneys were excised immediately and cut into small pieces. One piece was weighed and homogenised for biochemical assays. A second piece was kept at −80°C for mRNA extraction and evaluation of gene expression. The third piece was placed in neutral-buffered formalin for histological examination.

### 2.2. Kidney Weight Estimate

The relative kidney weight was estimated according to the following formula:

(1) $$\begin{array}{c} \text{relative kidney weight }=\frac{\text{left kidney weight }}{\text{body weight}}\;\times 100. \end{array}$$

### 2.3. Biochemical Parameters

#### 2.3.1. Serum Kidney Function Parameters

The serum levels of urea and creatinine were determined using kits supplied by Randox Laboratories (Crumlin, United Kingdom) according to the manufacturer's instructions.

#### 2.3.2. Preparation of Tissue Homogenates

Kidney tissues were homogenised in 50 mM tris-HCl (pH 7.4) to a final concentration of 10% (w/v). The obtained homogenates were centrifuged at 5,000 ×g for 10 min at 4°C. The supernatants were divided into aliquots and stored at −80°C until further use for biochemical analyses. The renal protein content was determined according to the method of Lowry et al. [9]. Bovine serum albumin (BSA) was used as a standard.

#### 2.3.3. Determination of Oxidative Stress Markers

The levels of malondialdehyde (MDA), an end product of lipid peroxidation (LPO), were determined using the thiobarbituric acid reactive substances method according to Ohkawa et al. [10]. Nitric oxide (NO) was estimated according to method described by Green et al. [11]. Superoxide dismutase (SOD) activity was measured according to the method described by Sun et al. [12]. Catalase (CAT) activity was measured according to the method described by Aebi [13]. Glutathione peroxidase (GPx) activity was measured according to the method described by Paglia and Valentine [14]. Glutathione reductase activity was measured according to the method described by Factor et al. [15]. Glutathione (GSH) quantitation was performed according to the method described by Ellman [16].

#### 2.3.4. Quantitation of Proinflammation Cytokine Levels

The renal levels of tumor necrosis factor-*α* (TNF-*α*) and interleukin-1*β* (IL-1*β*) were determined using ELISA kits purchased from Thermo Fisher Scientific (catalogue number: ERIL1B) and R&D Systems (catalogue number: RTA00), respectively. Analyses were performed according to the manufacturer's instructions.

#### 2.3.5. Determination of the Levels and Activity of Apoptotic Markers

The levels of the pro- and antiapoptotic proteins, Bcl-2 and Bax, were determined using ELISA kits purchased from Cusabio (catalogue numbers: CSB-EL006328RA and CSB-E08854r) and BioVision, Inc. (catalogue number: E4513) according to the suppliers' protocols, respectively. The activity of caspase-3 was determined using a calorimetric kit obtained from Sigma-Aldrich (catalogue number CASP3C).

#### 2.3.6. Real-Time Quantitative Polymerase Chain Reaction (RT-qPCR) Analysis

Total RNA from kidney tissue was extracted using the standard TRIzol® protocol (Invitrogen, Carlsbad, CA, USA). The obtained RNA was immediately converted to complementary DNA. The primer sequences used to determine the levels of nuclear factor (erythroid-derived 2)-like-2 factor (*Nfe2l2*) inducible nitric oxide synthase (*Nos2*), *IL-1β, TNF*-α*, Bax, Casp3,* and *Bcl2* gene expressions and are provided in Table 1 according to Abdel Moneim [17]. Power SYBR® Green Master Mix was utilised for RT-qPCR analysis. Glyceraldehyde-3-phosphate dehydrogenase (*Gapdh*) was used as the housekeeping gene, and its expression remained unaltered throughout the experiment. The fold changes in the examined genes were determined using the 2^−ΔΔCt^ method [18]. All experiments were performed in triplicate.

#### 2.3.7. Histopathological Examination

The kidney tissue was fixed in 10% neutral-buffered formalin for 24 h at room temperature, dehydrated, paraffinised, sectioned (5 *μ*m), and stained with hematoxylin and eosin for light microscopy examination. Images were captured using a Nikon microscope (Eclipse E200-LED, Tokyo, Japan) at a magnification of ×400.

### 2.4. Statistical Analysis

The results are expressed as means ± standard errors of the mean (*n* = 7). Comparisons between two groups were made using Student's *t*-test, and comparisons between three or more groups were made by one-way analysis of variance and *post hoc* Duncan's test using SPSS version 20.0.; *P* values <0.05 indicated statistical significance.

## 3. Results

### 3.1. Effect of RBR on Kidney Function Markers following CPF Exposure

**Figure 1:**
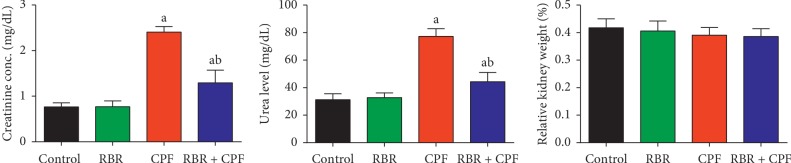
Protective effect of the red beetroot extract (RBR) on serum creatinine and urea and kidney index (relative kidney weight) in response to chlorpyrifos (CPF) exposure. The data are expressed as the mean ± SD for each experimental group (*n* = 7); ^a^*P* < 0.05 indicates a significant difference between treatment groups versus the control group. ^b^*P* < 0.05 indicates a significant difference compared with the CPF-exposed group.

After 4 weeks of 10 mg/kg CPF exposure, blood creatinine and urea levels, which are markers of kidney function, were significantly increased (Figure 1), indicating that CPF caused nephrotoxicity in male rats. CPF treatment also resulted in increased kidney index. Pretreatment with RBR 1 h prior to CPF administration reduced the increases in creatinine, urea, and kidney index values compared to those in rats only treated with CPF, suggesting that RBR protected against renal damage.

### 3.2. Effect of RBR on Redox Status in Kidney Tissue following CPF Exposure

**Figure 2:**
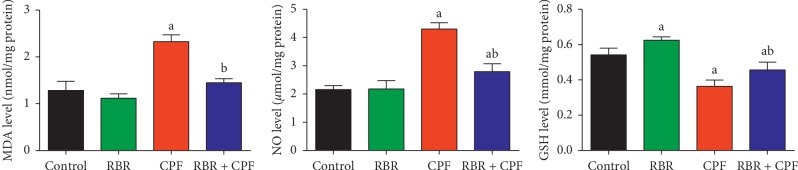
Protective effect of the red beetroot extract (RBR) against CPF exposure as demonstrated by decreased malondialdehyde (MDA) and nitric oxide (NO) levels and increased glutathione content (GSH) in renal tissue. ^a^*P* < 0.05 indicates a significant difference between treatment groups versus the control group. ^b^*P* < 0.05 indicates a significant difference compared between the CPF-exposed group.

**Figure 3:**
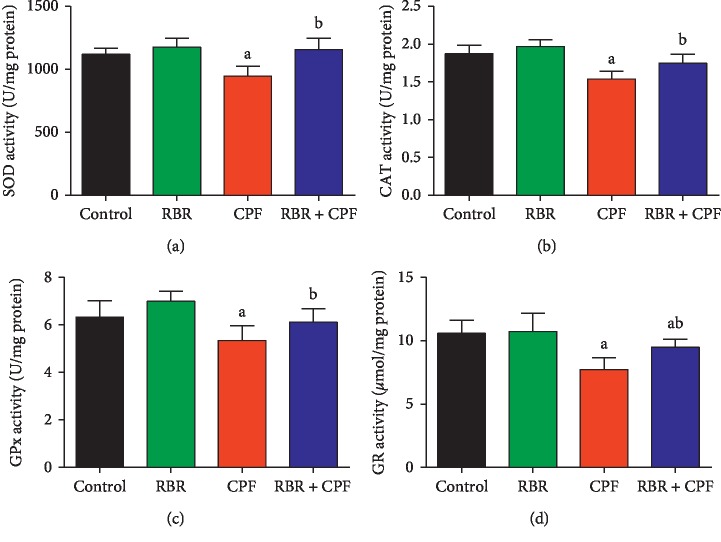
Protective effect of the red beetroot extract (RBR) against CPF-induced changes in (a) superoxide dismutase (SOD), (b) catalase (CAT), (c) glutathione peroxidase (GPx), and (d) glutathione reductase (GR) activity in renal tissue.

Because oxidative stress was a likely mechanism of CPF-induced nephrotoxicity, the oxidative stress markers including MDA, NO, antioxidant enzymes, and GSH were evaluated. Kidneys of rats treated with CPF had significantly increased (*P* < 0.05) MDA and NO levels, significantly decreased GSH content, and significantly decreased SOD, CAT, GPx, and GR activities compared to those in the control group. RBR pretreatment blocked CPF-induced changes in redox status, suggesting that RBR induced antioxidant effects in CPF-treated rat kidneys (Figures 2 and 3).

**Figure 4:**
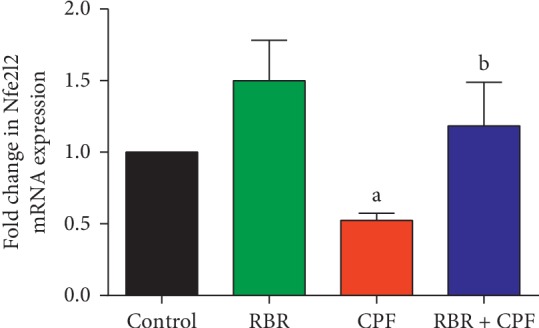
Protective effect of the red beetroot extract (RBR) against CPF-induced changes in *Nfe2l2* mRNA expression in renal tissue. Data are presented as the mean ± SD of three analyses normalised to *Gapdh* and presented as fold changes (log 2 scale) compared with the mRNA levels of the control and CPF-exposed groups.

Nrf2 is an important regulator of cellular resistance to xenobiotics. Nrf2 regulates the basal and enhanced expression of a variety of antioxidant response element-dependent genes to mitigate the physiological and pathophysiological effects of xenobiotic exposure. To investigate whether RBR induced antioxidant effects through Nrf2, *Nfe2l2* mRNA expression in kidney tissue was measured using qRT-PCR. *Nfe2l2* mRNA expression in kidney tissue was significantly downregulated in the CPF-treated rats compared to that in the control rats (Figure 4). RBR pretreatment resulted in significant upregulation of *Nfe2l2* mRNA expression in CPF-treated rats, demonstrating that RBR prevented nephrotoxicity in rats by enhancing *Nfe2l2* expression.

### 3.3. Effect of RBR on the Inflammatory Response in Kidney Tissue following CPF Treatment

**Figure 5:**
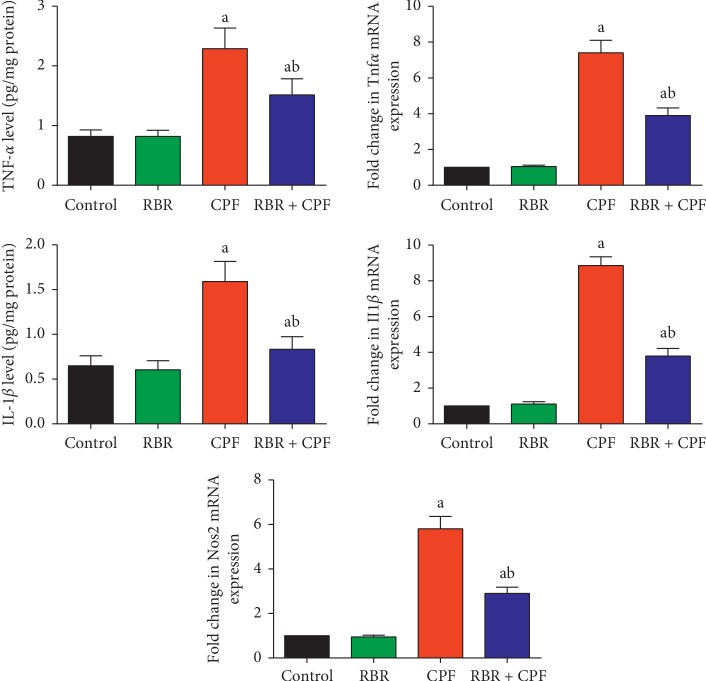
Protective effect of the red beetroot extract (RBR) against CPF-induced increases in the mRNA and protein expression of tumor necrosis factor-*α* (TNF-*α*), interleukin-1*β* (IL-1*β*), and inducible nitric oxide synthase (*Nos2*) in renal tissue. ELISA data are expressed as the mean ± SD for each experimental group (*n* = 7), whereas qRT-PCR data are presented as the mean ± SD of three analyses normalised to *Gapdh* and presented as fold changes (log2 scale) compared with the mRNA levels of the control and CPF-exposed groups. ^a^*P* < 0.05 indicates a significant difference between treatment groups versus the control group. ^b^*P* < 0.05 indicates a significant difference compared with the CPF-exposed group.

Transcriptional levels of the proinflammatory cytokines *Tnfα*, *IL-1β*, and *Nos2* were determined in kidney tissue of rats treated with CPF. As demonstrated by RT-qPCR, the mRNA expression of *TNF-α*, *IL-1β*, and *Nos2* was significantly upregulated in kidney tissue of rats treated with CPF compared to that in the control group (Figure 5). Furthermore, ELISA was used to confirm that the transcription-level results correlated to changes in translation. Our results showed that CPF treatment resulted in significant elevation in TNF-*α* and IL-1*β* protein levels compared to those in the control group. However, RBR pretreatment reduced CPF-induced up-regulation of *TNF-α*, *IL-1β,* and *Nos2* and prevented CPF-induced increases in TNF-*α* and IL-1*β* protein levels, suggesting that RBR exerted anti-inflammatory effects that could contribute to protection against CPF-induced nephrotoxicity.

### 3.4. Effect of RBR on Apoptosis-Related Proteins in Renal Tissue following CPF Exposure

**Figure 6:**
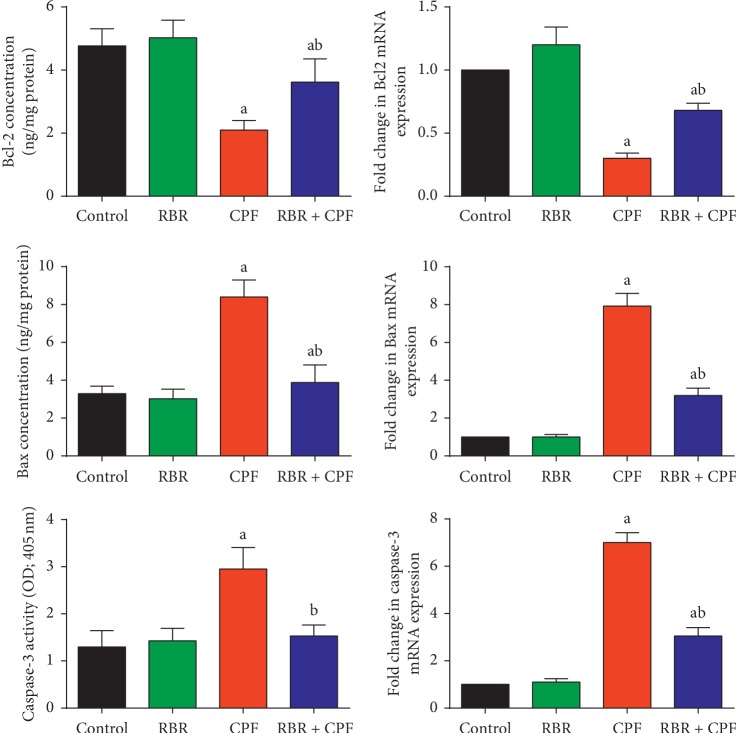
Protective effect of the red beetroot extract (RBR) against CPF-induced changes in the mRNA and protein expression of apoptosis-related proteins including Bcl-2, Bax, and caspase-3 in renal tissue. ELISA data are expressed as the mean ± SD for each experimental group (*n* = 7), whereas qRT-PCR data are presented as the mean ± SD of three analyses normalised to *Gapdh* and presented as fold changes (log 2 scale) compared with the mRNA levels of the control and CPF-exposed groups. ^a^*P* < 0.05 indicates a significant difference between treatment groups versus the control group. ^b^*P* < 0.05 indicates a significant difference compared with the CPF-exposed group.

To determine if apoptosis played a role in CPF-induced nephrotoxicity in rats, we measured Bcl-2, Bax, and caspase-3 mRNA and protein levels in kidney tissue. The proapoptotic markers Bax and caspase-3, and the antiapoptotic marker Bcl-2 were measured by qRT-PCR and ELISA. Our results showed that subchronic exposure to CPF significantly increased the mRNA and protein expression of Bax and caspase-3, and decreased the mRNA and protein expression levels of Bcl-2 compared to those in the control group. Pretreatment with RBR protected the renal tissue against CPF-induced nephrotoxicity by preventing CPF-induced changes in Bax, Bcl-2, and caspase-3 (Figure 6). Our results suggested that RBR increased antiapoptotic protein expression, thus exerting a protective effect against CPF-induced nephrotoxicity.

### 3.5. Effect of RBR on CPF-Induced Histopathological Alterations in Kidney Tissue

**Figure 7:**
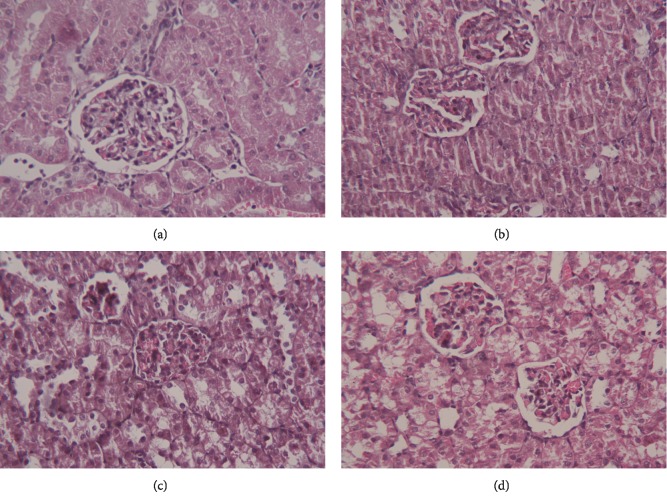
Protective effect of the red beetroot extract (RBR) against histopathological changes-induced by chlorpyrifos (CPF) exposure. (a) Control group, (b) RBR group, (c) CPF group, and (d) RBR-CPF group. Hematoxylin and eosin staining, magnification ×400.

Representative histopathological sections of the kidney tissue from control and the treatment groups are shown in Figure 7. The kidney tissue of the control rats and rats treated with RBR alone had normal kidney structure with normal renal tubules and glomeruli. CPF exposure for 28 days resulted in swelling of epithelial cells, oedema of the intertubular spaces, focal haemorrhage, inflammatory cell infiltration, vacuolisation, and development of intraluminal casts. Pretreatment with RBR minimised the pathological alterations induced by CPF exposure in kidney tissue.

## 4. Discussion

The kidney plays an important role in metabolism and excretion of chemicals and drugs, as it contains most common xenobiotic detoxifying enzymes [19]. Xenobiotic-induced renal injury is dependent on the chemical nature of the xenobiotic, the dose, and the duration of exposure. Using antioxidant agents to protect renal tissue against organophosphorus compound exposure has been previously reported [20]. The aim of the current study was to assess the potential nephroprotective role of RBR against CPF-induced renal injury. Exposure to CPF significantly increased the levels of serum creatinine and urea. Several physiological alterations have been observed following CPF exposure [21, 22]. Creatinine and urea are essential markers of kidney function in patients suffering from renal injury. Creatinine and urea are metabolic waste products mainly excreted from the body in the urine. Hence, increased levels of these markers following CPF exposure reflect renal dysfunction [23]. Increased creatinine in the serum reflects decreased glomerular filtration rate, while elevated urea indicates dysfunctional reabsorption [23]. Previous studies observed glomerular and renal tubular impairments in response to CPF exposure [24].

Interestingly, pretreatment with RBR significantly mitigated CPF-induced increases in serum creatinine and urea levels, demonstrating that RBR exerted renoprotective effects through maintaining membrane integrity and limiting the leaking of these biomarkers into the blood. Our findings agreed with those in a previous report El Gamal et al. [25] which showed that oral administration of the beetroot extract protected renal tissue against gentamicin-induced nephrotoxicity in rats, as determined by mitigation of serum creatinine and urea elevations.

Our study showed that CPF induced redox imbalance, as evidenced by increased MDA and NO levels, decreased GSH levels, and decreased activity of SOD, CAT, GPx, and GR in renal tissue. Oxidative stress has been suggested to be the primary mechanism of CPF-induced nephrotoxicity [21, 22]. MDA is formed by ROS-induced lipid peroxidation and is commonly used as a biomarker of oxidative stress. Increased MDA levels indicated damage to kidney tissue and altered membrane function [22, 26]. NO is a biological mediator involved in several physiological functions. Increased NO in response to CPF exposure suggests induction of nitrosative stress responses, likely due to upregulation of *Nos2*, the rate-limiting enzyme in NO synthesis [23]. GSH is a cellular tripeptide that acts as a potent scavenger of intracellular free radicals [27]. The marked decrease in renal GSH levels in response to CPF exposure was due to its consumption by scavenging of free radicals [23]. Depletion of the GSH pool also occurs through conjugation of GSH with electrophilic metabolites of CPF and inhibition of accumulation of MDA [28]. The enzymatic antioxidant enzymes SOD, CAT, GPx, and GR play fundamental roles in the elimination of reactive oxygen and nitrogen species which are responsible for cellular oxidative damage [29]. SOD metabolises superoxide radicals produced as a byproduct of increased metabolic activity in response to xenobiotics [30]. Deceased SOD activity indicates impairment of the renal antioxidant defence system against superoxide radicals [22]. CAT metabolises hydrogen peroxide, which is produced mainly in the mitochondria. Decreased CAT activity enhances accumulation of hydrogen peroxide, resulting in renal damage [22]. GPx is a selenium-containing antioxidant enzyme, which prevents decomposition of lipid hydroperoxides through reduction of hydroperoxides to alcohols. GPx also decreases free H_2_O_2_, thus providing cellular protection against oxidative stress [22]. GPx activity is dependent upon cellular GSH availability [22]. Therefore, we hypothesised that the observed decrease in GPx activity in response to CPF was due to decreased GSH in the renal tissue. Inactivation of these endogenous antioxidants in renal tissue following CPF treatment has been attributed to accumulation of cytotoxic free radicals in the kidney [20, 22]. In addition to disruption of the cellular antioxidant defence system, CPF significantly downregulated *Nfe2l2* mRNA expression in renal tissue. Nrf2 provides cellular protection against oxidative insults through increased expression of enzymes and proteins critical to the antioxidant response. Disturbances in Nrf2 signalling have been reported in several pathological conditions [31].

RBR treatment significantly mitigated the effects of CPF administration on *Nfe2l2* mRNA expression, GSH levels, and SOD, CAT, GPx, and GR activity, suggesting that RBR prevented CPF-induced damage through antioxidant mechanisms in the kidney. The antioxidant properties of RBR have been discussed in previous reports [32–34]. The antioxidant properties of RBR have been previously attributed to its rich betalain content, which triggers activation of Nrf2, resulting in enhancement of the expression of endogenous antioxidant enzymes [35]. In addition, RBR is a nitrate donor, resulting in the ability to scavenge reactive oxygen and nitrogen species including superoxide and hydrogen peroxide [36]. Indeed, RBR maintained antioxidant effectors at normal cellular levels despite oxidative challenge through upregulation of *Nfe2l2* mRNA expression.

Our results showed excessive release of TNF-*α* and IL-1*β* in response to CPF exposure, indicating that CPF induced inflammation in renal tissue. We also showed that CPF increased mRNA expression of *TNF-α* and *IL-1β* which was likely responsible for the increased levels of TNF-*α* and IL-1*β*. A previous study suggested that CPF induced oxidative stress, leading to inflammation, which includes increased production of TNF-*α* and IL-1*β* [23]. El-Sayed et al. [28] attributed the elevation in TNF-*α* and IL-1*β* to the overproduction of ROS and activation of nuclear factor kappa B (NF-*κ*B). Based on this attribution, we hypothesised that ROS scavengers/quenchers might effectively minimise CPF-induced inflammation in renal tissue. RBR exerted anti-inflammatory activity when administered prior to CPF as evidenced by decreased levels of TNF-*α* and IL-1*β*. El Gamal et al. [25] showed that RBR administration decreased inflammatory cell infiltration and significantly decreased the release of proinflammatory cytokines through the deactivation of NF-*κ*B in renal tissue following treatment with gentamicin. Moreover, betalains, the major active constituents in beetroot, were found to exert anti-inflammatory effects through inhibition of the expression of cyclooxygenase-2 *in vitro* [37].

Programmed cell death is considered a critical mechanism of CPF-induced toxicity. In the current study, CPF enhanced the apoptotic pathway in renal tissue as demonstrated by downregulation of Bcl-2 and upregulation of Bax and caspase-3. Bcl-2 is an essential regulator of apoptosis, and its overexpression is associated with suppression of ROS-induced apoptosis. In contrast, Bax and caspase-3 overexpression enhances cellular impairment and promotes proapoptotic signalling. A previous report showed that CPF exposure induced downregulation of Bcl-2 and upregulation of Bax and caspase-3, suggesting that CPF may trigger cell death via caspase-dependent mitochondrial pathways [38]. Initiation of apoptotic cascades in response to CPF has been also attributed to mitochondrial dysfunction and overproduction of ROS associated with the progression of oxidative stress [39, 40]. Furthermore, CPF has also been shown to activate apoptosis through upregulation of proapoptotic proteins in liver tissue [41]. Antioxidant treatment has been strongly linked with inhibition of chemical-induced apoptosis [19]. RBR coadministration with CPF exerted antiapoptotic activity through upregulation of Bcl-2 and downregulation of Bax and caspase-3. In addition, [25] showed that treatment with red beetroot increased the expression of Bcl-2 and decreased the expression of Bax and caspase-3 in gentamicin-treated rats.

## 5. Conclusion

We showed that CPF administration increased markers of kidney dysfunction, caused an imbalance between oxidative stress markers (MDA and NO) and antioxidant effectors (GSH, SOD, CAT, GPx, GR, and Nrf2), increased proinflammatory cytokine production, and induced apoptosis in renal tissue. However, RBR treatment prior to CPF administration reversed these changes in renal tissue through antioxidant, anti-inflammatory, and antiapoptotic mechanisms.

## Figures and Tables

**Table 1:** Primer sequences of genes analyzed in real-time PCR.

Name	Accession number	Forward primer (5′---3′)	Reverse primer (5′---3′)
*Gapdh*	NM_017008.4	AGTGCCAGCCTCGTCTCATA	GATGGTGATGGGTTTCCCGT
*Nfe2l2*	NM_031789.2	TTGTAGATGACCATGAGTCGC	ACTTCCAGGGGCACTGTCTA
*Nos2*	NM_012611.3	GTTCCTCAGGCTTGGGTCTT	TGGGGGAACACAGTAATGGC
*IL-1β*	NM_031512.2	GACTTCACCATGGAACCCGT	GGAGACTGCCCATTCTCGAC
*TNF-α*	NM_012675.3	GGCTTTCGGAACTCACTGGA	CCCGTAGGGCGATTACAGTC
*Bcl2*	NM_016993	ACTCTTCAGGGATGGGGTGA	TGACATCTCCCTGTTGACGC
*Bax*	NM_017059.2	GGGCCTTTTTGCTACAGGGT	TTCTTGGTGGATGCGTCCTG
*Casp3*	NM_012922.2	GAGCTTGGAACGCGAAGAAA	TAACCGGGTGCGGTAGAGTA

The abbreviations of the genes: G*apdh*, glyceraldehyde-3-phosphate dehydrogenase; *Nfe2l2*, nuclear factor (erythroid-derived 2)-like-2 factor; *Nos2*, inducible nitric oxide synthase; *IL-1β*, interleukin-1 beta; *TNF*, tumor necrosis factor; *Bcl2*: B-cell lymphoma 2; *Bax*, Bcl-2-like protein 4; *Casp3*, caspase-3.

## Data Availability

The data used to support the findings of this study are included within the article.
